# Molecular Characterization of ESBL-Producing Enterobacteriaceae in Northern Portugal

**DOI:** 10.1155/2014/782897

**Published:** 2014-02-13

**Authors:** Rúben Fernandes, Paula Amador, Carla Oliveira, Cristina Prudêncio

**Affiliations:** ^1^Ciências Químicas e das Biomoléculas (CQB) e Centro de Investigação em Saúde e Ambiente (CISA), Escola Superior de Tecnologia da Saúde do Porto (ESTSP), Instituto Politécnico do Porto (IPP), 4400-330 Porto, Portugal; ^2^CFBQ-Centro de Farmacologia e Biopatologia Química (U38-FCT), Faculdade de Medicina do Porto, Porto, Portugal; ^3^Departamento de Ciências do Ambiente, Escola Superior Agrária de Coimbra, Instituto Politécnico de Coimbra, Coimbra, Portugal; ^4^Departamento de Biologia Molecular, Instituto de Ciência Biomédicas de Abel Salazar (ICBAS), Universidade do Porto, Porto, Portugal; ^5^Centro Hospitalar da Universidade de Coimbra (CHUC), Coimbra, Portugal

## Abstract

Extended-spectrum **β**-lactamases (ESBLs) prevalence was studied in the north of Portugal, among 193 clinical isolates belonging to citizens in a district in the boundaries between this country and Spain from a total of 7529 clinical strains. In the present study we recovered some members of Enterobacteriaceae family, producing ESBL enzymes, including *Escherichia coli* (67.9%), *Klebsiella pneumoniae* (30.6%), *Klebsiella oxytoca* (0.5%), *Enterobacter aerogenes* (0.5%), and *Citrobacter freundii* (0.5%). **β**-lactamases genes blaTEM, blaSHV, and blaCTX-M were screened by polymerase chain reaction (PCR) and sequencing approaches. TEM enzymes were among the most prevalent types (40.9%) followed by CTX-M (37.3%) and SHV (23.3%). Among our sample of 193 ESBL-producing strains 99.0% were resistant to the fourth-generation cephalosporin cefepime. Of the 193 isolates 81.3% presented transferable plasmids harboring *bla*_ESBL_ genes. Clonal studies were performed by PCR for the enterobacterial repetitive intragenic consensus (ERIC) sequences. This study reports a high diversity of genetic patterns. Ten clusters were found for *E. coli* isolates and five clusters for *K. pneumoniae* strains by means of ERIC analysis. In conclusion, in this country, the most prevalent type is still the TEM-type, but CTX-M is growing rapidly.

## 1. Introduction

Extended-spectrum *β*-lactamases (ESBLs) are enzymes that confer resistance to aztreonam, cefotaxime, ceftazidime, and related oxyimino-*β*-lactams as well as to other penicillins and cephalosporins but are inhibited by clavulanic acid [1]. ESBL-producing Enterobacteriaceae were first reported in Europe in the 1980s and have since become a worldwide problem [2]. This has resulted in increased morbidity, mortality, and cost in treating the infections they cause [3]. The first ESBL were mutants of the TEM and SHV plasmid-mediated penicillinases with one or more amino acid substitutions. The mutations confer resistance to all oxyimino-cephalosporins but not *α*-methoxy-cephalosporins (cephamycins) or carbapenems by causing enlargement of ESBL active site, which allowed the deflection of the oxyimino group diminishing the attack efficiency on the *β*-lactam ring [4]. TEM and SHV present to date over 200 members known (http://www.lahey.org/studies). Another ESBL group includes the CTX-M enzymes that are organized in five major CTX-M groups: 1, 2, 8, 9, and 25 [5]. The CTX-M comprises a rapidly growing family distributed both over wide geographic areas and among a wide range of bacteria of clinical significance and is becoming more prevalent than its ancestors TEM and SHV [6]. Several studies have been reported in Iberian Peninsula describing the genetic and clinical environments of ESBL occurrence [7–12]. Here we report the molecular and antimicrobial susceptibility profile of the ESBL-producing clinical isolates, found in the Portuguese occidental coast in the boundaries between the two countries, Portugal and Spain. For this task we used methods of molecular typing which have been developed for the identification of the *β*-lactamases *bla *genes from both hospitalized and nonhospitalized patients for a period of two years.

## 2. Materials and Methods

### 2.1. Bacterial Strains, Identification, and Susceptibility

A total of 7529 clinical strains were included in the study. All isolates were gently provided from Clinical Pathology Laboratories and belong to patients samples recovered from September 2008 to August 2010 in the northern occidental coast of the Portuguese territory known as Minho (Portugal). This region comprises several populations of the north of Portugal and some at the boundaries with Galicia (Spain). Microbial identification and preliminary antimicrobial susceptibility were determined according to *Clinical and Laboratory Standards Institute* guidelines [13]. ESBL production was confirmed by the ellipsoid method, using two *E*-test Strips (AB Biodisk, Sweden), namely, TZ/TZL (ceftazidime and clavulanic) and CT/CTL (cefotaxime and clavulanic).

### 2.2. Conjugation Experiments

Transmissibility of resistance was studied by matting clinical isolates with the *E. coli* J53 Azi^R^ (azide resistant) on Trypticase soy broth (TSB) according to a method described previously [14].

### 2.3. Analytical Isoelectric Focusing (IEF)

Crude preparations of *β*-lactamases from clinical strains transconjugants were obtained by sonication in phosphate buffer, pH 7.0, as described previously [15]. Briefly crude extracts were concentrated and a nitrocefin solution was added. The color change from yellow to red indicates a positive *β*-lactamase production. The sample then was run on an IEF minigel, pH 3–10, for 30 minutes. *β*-lactamases isoelectric point (pI) was determined by pouring molten 3% agarose containing nitrocefin over the gel and comparing the bands to standards run on the same gel. The *β*-lactamase standards used were TEM-1, pI 5.4, SHV-5, pI 8.2, and CTX-M-14, pI 8.1.

### 2.4. Genetic Molecular Characterization of *bla *Genes and Typing

A single colony of each transconjugant was left to grow for 16 h on MacConkey agar and was placed in 200 *μ*L sterile water in a 1.5 mL microtube. Each tube was heated in a microwave oven at 600–700 W for 2 min to burst the cells and release their DNA. PCR was performed as described previously [15]. The recovered bands from agarose gels were cloned for further sequencing. The nucleotide sequences of both ends of the insert were determined with M13 sequencing primers specific for the cloning vector [16]. ERIC profiles were analyzed with software FPQuest version 4.5, Fingerprinting II (Bio-Rad Laboratories, CA, USA).

## 3. Results

The prevalence of ESBL-producing strains in the north Portuguese territory was 2.6% (*n* = 193). The most frequent ESBL-producing organism was *E. coli* (67.9%, *n* = 131), followed by *K. pneumoniae* (30.6%, *n* = 59), *K. oxytoca* (0.5%, *n* = 1), *E. aerogenes *(0.5%, *n* = 1), and *C. freundii* (0.5%, *n* = 1) as shown in Table 1. ESBL-producing strains were isolated from urine (*n* = 127), sputum (*n* = 42), bronchoalveolar lavage (*n* = 14), bloodstream (*n* = 7), and ascitic fluid (*n* = 3).

PCR studies allowed detecting several ESBL enzyme types. TEM enzymes were the most frequent ESBL types (40.9%), followed by CTX-M (37.3%) and finally SHV (23.3%). Sequencing confirmed ESBL and allowed identifying the enzyme variant. TEM-52 and TEM-24 were the most frequent TEM types, 20.2% (*n* = 39) and 12.9% (*n* = 25), respectively. Members of the TEM-10 (*n* = 8) and TEM-116 (*n* = 4) were also detected. Within CTX-M family, the CTX-M-9 group is more prevalent than the CTX-M-1 group (58.3% against 41.6%). CTX-M-9 group was represented by CTX-M-9 (*n* = 26, 36.1%) and CTX-M-14 (*n* = 16, 22.2%). In the CTX-M-1 group, CTX-M-15 was most frequent type (*n* = 24, 33.3%), followed by CTX-M-1 (*n* = 4, 5.5%), CTX-M-3 (*n* = 1, 1.3%), and CTX-M-32 (*n* = 1, 1.3%). The SHV enzymes occurred only in 23.3% of all ESBL-producing organisms. Within this type, the most frequent was the SHV-12 variant (*n* = 24, 53.3%), followed by SHV-5 (*n* = 17, 37.8%) and finally SHV-2 (*n* = 4, 8.8%). Some isolates coproduced more than one ESBL type: TEM-52/CTX-M-14 (*n* = 1), TEM-116/CTX-M-14 (*n* = 1), and TEM-116/CTX-M-15 (*n* = 1).

Regarding antibiotic susceptibility the present study shows that ESBL-producing strains were extremely resistant to cefepime (99.0%) and susceptible to carbapenems (100%).

In what concerns to interspecific genetic similarity, it was observed a high genetic diversity. It was possible to define 10 clusters (A to J) for *E. coli* based on Pearson's correlation coefficient in PCR-ERIC based profile. For *K. pneumoniae* 5 different clusters (K to O) were defined for the PCR-ERIC (data presented as Supplementry Material available online at http://dx.doi.org/10.1155/2014/782897).

## 4. Discussion

As reported in previous Portuguese [9–12, 14, 15, 17–19], Spanish [8, 20, 21], and other European [6] studies *E. coli* and *K. pneumonia* are the species where ESBL is the most frequently identified. In this study *E. coli* was the most frequent (*n* = 131) organism expressing ESBL phenotypes, more than twofold of the *K. pneumonia* (*n* = 59), the second most frequent.

**Figure 1:**
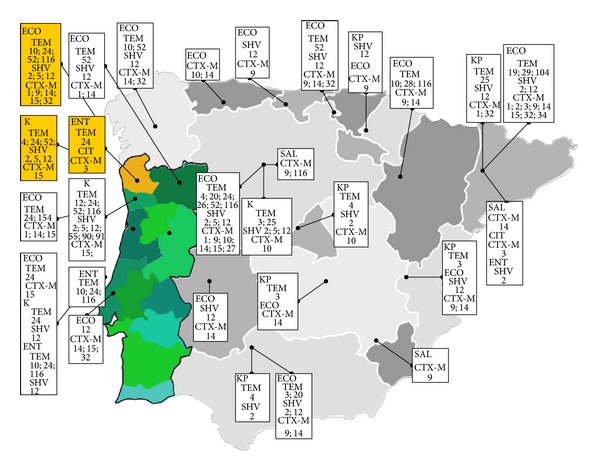
Occurrence of ESBL-TEM, ESBL-SHV, and ESBL-CTX-M types in the north of Portugal. The present study, territory, Minho, is represented at the map in yellow. Other regions either from Portugal vs Spain are represented in different shades of green and gray or, respectively. Data was collected from the present study (yellow) and from several studies in these two countries [8–12, 14, 15, 17–26]. [ECO:*Escherichia coli*; ENT:*Enterobacter spp*.; KP:*Klebsiella pneumoniae*; K:*Klebsiella spp*.; SAL:*Salmonella enterica*].

Regarding the high diversity ESBL types obtained in our study, we find it interesting to compare with neighbor regions, such as Douro Litoral located at south of Minho, Trás-os-Montes e Alto Douro, eastern, and the Spanish province of Galiza, located at north of Minho (Figure 1).

From this evaluation and regarding ESBL-TEM types data suggests that TEM-10, TEM-20, TEM-26, and TEM-116 from *E. coli* and TEM-4 from *K. pneumoniae* were present not only in the region analyzed in the present study (Minho) but also in other locations of Iberian Peninsula. *E. coli* strains producing SHV-2 and SHV-5 were also found not only in Minho, Portugal, but also in other regions of Iberian Peninsula [8].

Regarding CTX-M types, it seems that CTX-M-14 is widespread within the northwestern Iberian Peninsula. *K. pneumoniae* harboring a CTX-M-15 was described for the first time in Portugal in 2005 [22] in Lisbon area, but it is also found in the north of Portugal in this study and Douro [9]. The high frequency of CTX-M-15 enzymes found in the present work is in agreement with the major studies worldwide that report the emergence of CTX-M-15 producing *E. coli* as new threat [21].

Cefepime presents, in this study, a surprisingly low activity against ESBL-producing microorganisms. In our sample only two *K. pneumoniae* harboring SHV-2 ESBLs were susceptible to cefepime. All the other clinical isolates 99.0% (*n* = 191) expressing the ESBL phenotype were resistant to cefepime. It seems interesting that a recent study showed that cefepime was successfully administrated to three patients (two females and one male) with ages between 47 and 87 years old carrying a gram-negative ESBL positive strain [27]. Nevertheless other studies worldwide start to describe the emergence of high resistance among ESBL gram-negative producers [12, 28, 29].

Regarding the age of the patients, 82% of those infected with an ESBL-producing pathogen are more than 60 years old. This finding is in accordance with a recent work that states that age over 65 years old is a risk factor for *β*-lactamase-mediated resistance to oxyimino-*β*-lactams in patients infected with enterobacteria [30].

Finally, considering the genetic relatedness of 191 isolates studied, by ERIC analysis, it is possible to propose 10 clusters for *E. coli* and 5 clusters for *K. pneumoniae. *This finding suggests a pronounced genetic diversity among ESBL-producing clones. Similar studies in southern Portugal considering simply ESBL-producing* K. pneumoniae* isolates have also demonstrated this extraordinary genetic diversity [31].

Furthermore, several studies suggest that in Portugal the presence of ESBL genes not only in clinical context but also in the environment, foodstuff, food-producing animals, selvage animals, waters (wasters and for human consumption). In general these studies show an increasing concern for these matters, an increasing of antibiotic resistance genes among other environments not associated with healthcare, and uncontrolled use of antibiotics with prolonged exposure often resulting in bacterial resistance [32–38]. Interestingly, regarding this matter, members of the team have identified three new TEM variants, within water and foodborne enterobacterial pathogens (TEM-179, TEM-180, and recently TEM-201) but not within the clinic isolates [37].

## 5. Conclusion

In summary, we reported the high biochemical and genetic diversity of ESBL enzymes occurring in Portugal. In this country, the most prevalent type is still the TEM type, but CTX-M is growing rapidly. Similarly high genetic diversity among ESBL-encoding strains is also observed by means of ERIC that allowed detecting distinct phylogenetic relationships between the different Enterobacteriaceae isolates.

The present study also provides strong evidence that resistance to fourth-generation cephalosporins is a matter of major concern in our country. The emergence of ESBL producers resistant to cefepime in Portugal may indicate the uncontrolled use of cephalosporins and that is an issue that we strongly believe is urgent to investigate.

## Supplementary Material

Supplementary material: Dendograms regarding interspecific genetic similarity, by means of ERIC-PCR, using Pearson's correlation coefficient. Figure A – 10 clusters (A to J) were defined for Escherichia coli. Figure B – 5 clusters (K to O) were defined Klebsiella pneumoniae.

## Figures and Tables

**Table 1:** Characterization of ESBL-producing strains.

ESBL	IEF (pI)	Microorganism (number of isolates)	Conjugation (% positive)	Resistance phenotype (% nonsusceptible: I + R)						ERIC types patterns*
				*β*-Lactams			Non-*β*-lactams			
				CEP	FOX	CARB	CIP	GEN	SXT	
TEM-4	5.9	*E. coli* (2) *K. pneumoniae* (1)	100 100	100 100	50 0	0 0	100 100	50 100	100 0	A L
TEM-10	6.0	*E. coli* (8)	87.5	100	25	0	87.5	0	75	B, D, H
TEM-24	6.5	*E. coli* (12) *K. pneumoniae* (11) *K. oxytoca* (1) *E. aerogenes* (1)	83.3 81.8 100 100	100 100 100 100	50 20 100 0	0 0 0 0	75 72.7 100 0	8.3 50 0 100	8.3 63.6 100 100	C, G, H, I K, L, O n.a. n.a.
TEM-52	6.0	*E. coli *(27) *K. pneumoniae* (12)	81.4 100	100 100	36.4 8.4	0 0	84.6 91.6	53.8 83.3	42.3 33.3	A, B, E, F, J N, O
TEM-116	5.4	*E. coli* (*n* = 4)	100	100	50	0	75	75	0	F
SHV-2	7.6	*K. pneumoniae* (3) *E. coli *(1)	100 100	33.3 0	33.3 0	0 0	66.7 100	66.7 100	100 100	L A
SHV-5	8.2	*K. pneumoniae* (11) *E. coli *(6)	72.7 83.3	100 100	27.3 66.7	0 0	63.6 100	72.7 100	27.3 0	L, N
SHV-12	8.2	*K. pneumoniae* (18) *E. coli *(6)	83.3 100	100 100	27.7 33.3	0 0	61.1 100	94.4 100	77.8 66.7	K, M, N, O C, D, E
CTX-M-1	6.3	*E. coli *(1)	100	100	25	0	100	100	0	E
CTX-M-3	8.4	*C. freundii* (1)	0	100	0	0	0	100	0	n.a.
CTX-M-9	8.1	*E. coli *(26)	92.3	100	26.9	0	96.2	84.6	3.8	A, C, D, G, I, J
CTX-M-14	8.1	*E. coli *(16)	68.8	100	12.5	0	100	75	0	D, F, H, J
CTX-M-15	8.6	*E. coli *(21) *K. pneumoniae* (3)	85.7 100	100 100	38.1 100	0 0	100 100	90.5 100	14.3 100	A, C, E, I, J N
CTX-M-32	9.0	*E. coli *(1)	100	100	0	0	100	100	0	D

ESBL: extended-spectrum *β*-lactamase; IEF: isoelectric focusing; pI: isoelectric point; I: intermediate; R: resistant; n.a.: not applicable. Antimicrobials: CEP: cefepime; FOX: cefoxitin; CARB: carbapenems (imipenem and meropenem); CIP: ciprofloxacin; Gen: gentamicin; SXT: combination of trimethoprim and sulfamethoxazole.

*Supplementary Material.
