# Supplementary material for: Molecular Characterization of ESBL-Producing Enterobacteriaceae in Northern Portugal
Source: ScientificWorldJournal. 2014 Feb 13;2014:782897. doi: 10.1155/2014/782897 (PMC3950362; doi:10.1155/2014/782897)
Supplement: Supplementary file 1 — Supplementary material: Dendograms regarding interspecific genetic similarity, by means of ERIC-PCR, using Pearson's correlation coefficient. Figure A – 10 clusters (A to J) were defined for Escherichia coli. Figure B – 5 clusters (K to O) were defined Klebsiella pneumoniae. [file 782897.f1.pdf]

Molecular characterization of ESBL-producing Enterobacteriaceae in the Northern Portugal

SUPPLEMENTAL MATERIAL

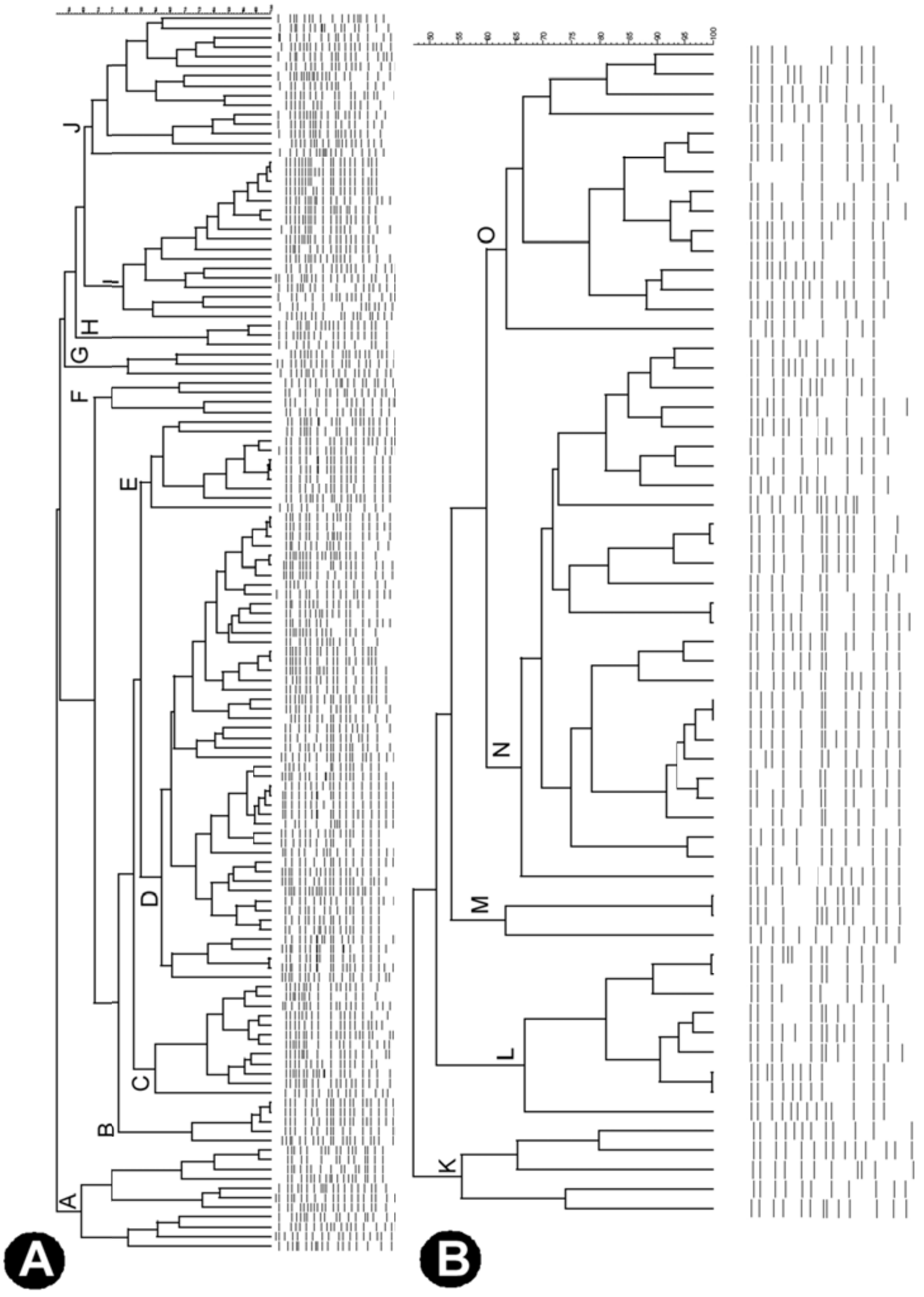

Figure: Enterobacterial repetitive intergenic consensus (ERIC) patterns of A: *Escherichia coli* and B: *Klebsiella pneumoniae*. This image shows high diversity among extended-spectrum beta-lactamases. Clusters A to H refer to ESBL-encoding *E. coli*. Clusters K to O refer to ESBL-encoding *K. pneumoniae*.
